# Development and Implementation of Workshops to Optimize the Delivery of Vaccination Services in Community Pharmacies: Thinking beyond COVID-19

**DOI:** 10.3390/pharmacy11040129

**Published:** 2023-08-13

**Authors:** Arnaud Lavenue, Isabelle Simoneau, Nikita Mahajan, Kajan Srirangan

**Affiliations:** 1 Toc Toc Communications, 104-7030 Rue Marconi, Montréal, QC H2S 3K1, Canada; isimoneau@toctoccommunications.com (I.S.); nikita.mahajan@hotmail.com (N.M.); kajan.srirangan@mail.concordia.ca (K.S.); 2 School of Pharmacy, University of Waterloo, 10A Victoria Street S., Kitchener, ON N2G 1C5, Canada

**Keywords:** community pharmacists, pharmacy, pharmacy practice, practice change, vaccines, immunization services, workshop, Canada, Québec

## Abstract

Vaccines are widely recognized as the most economically efficient strategy to combat infectious diseases. Community pharmacists, being highly accessible healthcare professionals, have the potential to significantly contribute to the promotion and facilitation of vaccination uptake. In Canada, the jurisdiction of healthcare falls under provincial legislation, leading to variations in the extent of pharmacist practice throughout the country. While some pharmacists in Canada already functioned as immunizers, Québec pharmacists gained the authority to prescribe and administer vaccines in March 2020 amidst the COVID-19 pandemic. Our workshop aimed to equip pharmacists in Québec with the necessary guidance to optimize vaccinations, emphasizing the importance of maintaining and expanding immunization services beyond influenza and COVID-19 vaccines in the future. During the workshop, pharmacists had the opportunity to exchange valuable insights and best practices regarding workflow optimization, identifying areas for improvement in competency, effectively reaching vulnerable population groups, and integrating allied team members into their practice. Participants were also asked to develop a plan of action to help implement practice change beyond the workshop. Interactive workshops centered around discussions like these serve as catalysts for advancing the pharmacy profession, uniting professionals with a collective aim of enhancing patient care.

## 1. Introduction

In recent years, there has been a notable increase in the engagement of pharmacists in delivering collaborative patient care. The United States, the United Kingdom, and several other countries around the globe exemplify this trend by fostering greater participation in primary care through interdisciplinary collaboration [1,2,3,4,5,6,7]. So far, pharmacists as prescribers have shown comparable efficacy outcomes when compared to usual medical care prescribing in the management of hypertension, cholesterol, chronic pain, diabetes, and other chronic disease states [8,9,10,11].

As the demand for healthcare services intensifies in Canada, pharmacists hold a unique position to address significant challenges within the Canadian healthcare landscape. One prominent issue is the limited access to care, since a considerable number of Canadians do not have a consistent primary care provider. For patients with a family physician, the issue then becomes that of time, as recent survey results show that only 39.9% of Canadians with family physicians are able to book same day appointments [12,13]. The provision of timely treatment plays a critical role in patient care, as delays in accessing treatment can have far-reaching psycho-social consequences. Patients may experience heightened levels of worry, stress, and anxiety, along with physical discomfort, placing strain on their families and friends and contributing to a decline in their overall health [14]. To enhance healthcare accessibility, decision-makers in Canada are currently reassessing the roles of healthcare providers, including nurses, physician assistants, and pharmacists, in the delivery of patient care, with the aim of expanding their contributions [15,16,17,18,19,20,21,22,23,24].

Nationwide, pharmacists have always been an important member of Canada’s healthcare system. Nearly 95% of Canadians live within a 5 km radius of a pharmacy. The geographic proximity to patients ideally makes pharmacists an avenue to increase accessibility of healthcare services [25]. Moreover, 55% percent of Canadians see their pharmacists ten times more often than their primary care physician [16]. Pharmacists, being highly trusted healthcare providers, frequently serve as the initial point of contact for many Canadians seeking healthcare services. Moreover, as the profession shifts from a conventional dispensing model to a more clinical focus, regulatory changes have been implemented across all provinces to enable pharmacists to assume a more active role in patient care [16,26].

In Canada, the scope of prescribing, administering, and monitoring drug therapies by community pharmacists varies due to healthcare regulation being governed at the provincial/territorial level [27,28]. In addition, the extent to which each are remunerated by public funds or private payers varies across jurisdictions. Professional services range from medication reviews, minor/common ailment management, pharmacist renewing for existing prescriptions, and smoking cessation counselling [16]. In addition to medication and chronic disease management, pharmacists’ scope of practice also includes the delivery of vaccination services. Whether acting as an educator by providing recommendations on vaccines, serving as a facilitator by incorporating other nurse immunizers in their practice to enable access, or being an administrator as an immunizer themselves, pharmacists have an established identity in immunization initiatives [29]. While some pharmacists are already functioning as immunizers in other Canadian provinces, due to provincial self-regulation, Québec pharmacists only recently gained the authority to prescribe and administer vaccines in March 2020 amidst the COVID-19 pandemic [27,30]. Prior to the pandemic, Québec pharmacists contributed to vaccination efforts by promoting vaccinations, providing counselling, and hosting onsite nurses to administer the vaccines [27].

In Canada, various pharmacy associations offer training and education on activities related to vaccine administration, including vaccine preparation, documentation, and addressing administration errors. However, these programs lack a specific emphasis on the practical aspects of day-to-day implementation, challenges related to execution, vaccination promotion, integration into daily practice, and the significance of collaborative teamwork among pharmacy assistants, nurses, and pharmacists. With the recent expansion of scope in Québec, pharmacists expressed a desire to enhance their proficiency in delivering vaccination services more effectively. They also raised concerns about the operational complexities associated with implementing and integrating these new services into their existing daily responsibilities. To address these needs and concerns, in our previous study, we conducted a series of accredited workshops focused on facilitating discussions about the perceived challenges in establishing a pharmacy-led vaccination clinic and optimizing their current vaccination services. These workshops were particularly relevant as nurses had previously led the vaccination services [31]. By engaging in active peer-to-peer discussions, pharmacists had the opportunity to exchange valuable insights regarding best practices for delivering effective vaccination services. They collectively identified shared clinical competency gaps related to adult vaccinations, explored strategies for enhancing patient communication skills, and devised approaches to specifically target vulnerable population groups [31]. In light of the positive feedback received from participants regarding the introduction of pharmacist-led immunization services, many participants felt that an extended standalone workshop was required in order to provide directions for maintaining and expanding future immunization services beyond influenza and COVID-19 vaccines.

**Figure 1:**
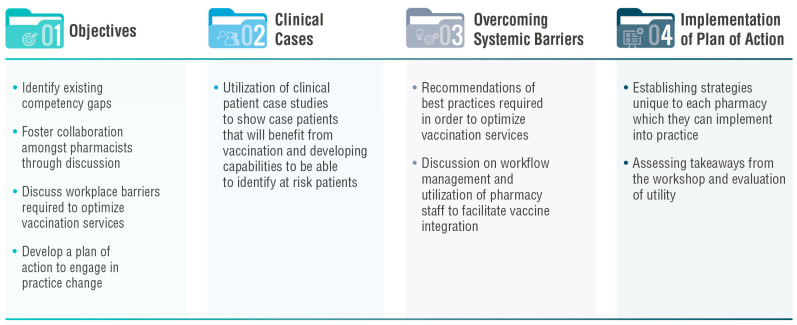
The workshop had four major objectives: (1) identifying competency gaps, (2) fostering peer-to-peer exchange, (3) discussing workplace barriers and (4) developing a plan of action (POA) to engage in practice change.

Herein, we report on the results of our most recent workshop, which sought to equip pharmacists with the skills to optimize delivery of vaccination services in their community by discussing key challenges including the identification of patients who will benefit from vaccinations, contribution of pharmacy staff, and integration of vaccination into pharmacy workflow. Pharmacists throughout the province who completed these workshops were awarded a continuing education unit (CEU) credit by the Ordre des pharmaciens du Québec (OPQ). As shown in Figure 1, the workshops had four overarching objectives: (1) identifying existing competency gaps of the pharmacists, (2) fostering peer-to-peer exchange, (3) discussing workplace barriers required to optimize immunization services (beyond COVID-19 vaccines) and (4) developing a plan of action (POA) to engage in practice change.

## 2. Methods

### 2.1. Participant Recruitment

To recruit pharmacists for these workshops, we reached out to professional services groups and sponsors that focused on pharmacists engaged in vaccination and those who were seeking to enhance their immunization services. Candidates were contacted via purposeful sampling via email and/or telephone. The resulting group comprised pharmacists from diverse backgrounds, with varying levels of interest in vaccination services, hailing from different geographical locations, and various chains and banners of high and low prescription volume. Moreover, the participants represented a wide range of work experience. It is important to note that we did not collect additional demographic information, such as age, race, or preferred gender identity, from the participating pharmacists.

### 2.2. Workshop Development

A series of 9 round table discussion-based workshops were held with the groups of 8–12 pharmacists who were in charge of clinical pharmacy services. Each workshop was banner-specific to ensure open discussions among participants. These workshops served two purposes: (1) We first sought to identify the challenges that participants felt were preventing them from optimizing vaccination services in their community pharmacy practice. These discussions were intended to capture the pharmacists’ perceived challenges in this new role. After identifying these challenges, we then sought to facilitate best practice discussions amongst peers to identify potential solutions. (2) These discussions were also meant to serve as a guide for the development of a POA specific to each pharmacy’s unique landscape. The intent of the POA was to create concrete direction that would facilitate further capacity in delivering vaccination services and optimize workflow practices for expansion to additional vaccines.

### 2.3. Workshop Evaluation

After concluding the workshop, pharmacists were requested to fill out an anonymous evaluation questionnaire that comprehensively assessed various aspects of the training session. Since all participants were French speaking, the questionnaire was written and completed in French. The evaluation questionnaire employed a 5-point Likert scale, encompassing six questions, with response options ranging from “low” to “very high”. The English translation of the questionnaire is available in Supplementary Material. Moreover, in the free-text portion of the evaluation, pharmacists were able to provide qualitative assessments on the strengths and limitations of the workshop. Finally, participants were also invited to participate in a follow-up discussion 2 weeks after the event to evaluate implementation of their POA. The discussion involved participants highlighting what was going well with their implementation of their personalized POA and encountered challenges thus far to facilitate ongoing uptake of this POA. The analysis of free-text responses followed a thematic approach, and the calculation of word frequency was conducted using the qualitative data management tool Taguette (www.taguette.org/ (accessed 20 March 2023)) [32].

## 3. Results and Discussion

In recent years, the field of pharmacy has undergone significant transformations to adapt to the evolving societal demands. Presently, pharmacists possess the necessary training and education to offer comprehensive medication management, conduct patient assessments, identify, and resolve drug therapy issues, collaborate with other healthcare professionals to develop care plans, and optimize treatment outcomes for patients. Numerous established pieces of evidence highlight the mutual advantages for both patients and pharmacists when the full extent of pharmacists’ training and education is effectively utilized [33].

Pharmacists have emerged as a crucial component of the immunization workforce, and research demonstrates that patients highly value the convenience and accessibility of pharmacy services, resulting in a high degree of patient satisfaction [34]. However, given Canada’s decentralized pharmacy regulatory environment, there remains heterogeneity on vaccines a pharmacist can prescribe and/or administer, if any, to whom, and under what funding model [30]. Considering Québec’s recent changes regarding the scope of pharmacists as immunizers, our series of workshops engaged pharmacists in practice discussion to identify challenges and develop solutions required for the continued success and expansion of pharmacy-based vaccination efforts.

**Figure 2:**
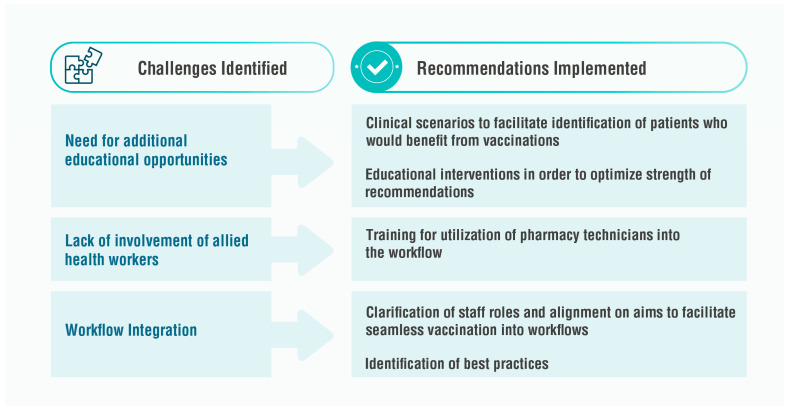
Three major challenges that were identified were (1) the need for additional educational opportunities, (2) managing allied healthcare workers (e.g., pharmacy assistants and nurses), and (3) effectively managing workflow and scheduling. Recommendations identified to tackle these challenges include development of clinical scenarios and educational interventions, better training of pharmacy assistants and other support staff and clarification of their roles, and identification of best practices from other pharmacies and pharmacists.

This workshop highlighted three key areas where participants identified competency gaps: (1) the need to improve skills in identifying vulnerable populations and offering evidence-based vaccination advice, (2) enhance the integration of allied healthcare workers, including pharmacy assistants and nurses, within the workflow, and (3) optimize workflow and scheduling practices for improved efficiency of vaccination services (Figure 2).

When addressing the first competency gap in the workshop, all respondents revealed that presently they had not been making proactive recommendations for immunizations. While all participants acknowledged the significance of providing additional vaccines to eligible individuals, some had not actively engaged in co-promoting vaccinations. Co-promoting involves offering additional vaccines, such as pneumococcal or shingles vaccines, to eligible patients alongside influenza vaccines.

Multiple barriers exist in relation to adult vaccinations, including the absence of a mandate for immunization as seen with children’s vaccinations [35]. This is of concern as most adults do not undergo a routine assessment by their care providers for vaccination status. Further issues arise as most adults receive care from differing providers or specialists, which can result in a lack of coordinated care and leave many patients unaware of their immunization status. Furthermore, vaccinations such as pneumococcal are potentially complex due to guideline changes based on the patients’ age, chronic conditions, and previous pneumococcal vaccination status [36]. A healthcare provider’s strong recommendation is one of the most influential factors positively affecting vaccination uptake. [37]. Nevertheless, our discussions uncovered that most vaccinations performed at the pharmacy occur when patients specifically request them. This indicates that engagement in adult vaccinations is passive and driven by patient initiative, potentially leading to missed chances for identifying other eligible patients. Recognizing that most participants indicated that they desired additional educational opportunities, specific resources and case-based examples were developed to help improve clinical knowledge and enable pharmacists to identify vulnerable population groups that can be recommended for vaccinations. By considering invasive pneumococcal disease as a reference condition, we created diverse patient profiles encompassing underlying pathologies or conditions that could potentially benefit from pneumococcal vaccination. Enhancing knowledge in this area enables practitioners to actively engage in discussions and provide comprehensive care to their patients [38]. Vaccine hesitancy also poses a significant obstacle to adult vaccination uptake. It involves the reluctance or refusal to receive readily available vaccines and is influenced by various demographic factors, including race/ethnicity, religion, and socioeconomic status [39]. In our workshops, all pharmacists recognized vaccine hesitancy as a persistent public health concern that has been underscored by the COVID-19 pandemic, and has implications for future pandemics, epidemics, and other vaccines. At the same time, while holding pro-vaccine viewpoints, pharmacists emphasized their commitment to respecting patients’ autonomy in making vaccination decisions. They firmly believed in their patients’ right to choose whether to be vaccinated or not. Nonetheless, they acknowledged that these interactions presented an opportunity to offer up-to-date information and dispel misconceptions about vaccine safety, ultimately playing a role in educating patients.

The second identified competency gap pertained to collaborating with allied health workers, particularly the utilization of pharmacy assistants. Pharmacy assistants play a crucial role in supporting pharmacists who face challenges in finding time to deliver additional clinical services amidst the dispensing workflow. They serve as valuable resources for optimizing patient care [40]. In our workshop, most respondents indicated the need to train pharmacy assistants to identify eligible or at-risk individuals to optimize their vaccination services. Without clear goals and shared clinical aims, a lack of staff involvement and communication can adversely affect patient outcomes and result in missed opportunities to educate patients [41]. Pharmacy assistants, being the first members of the team to interact with patients, have a significant opportunity to identify eligible individuals for vaccination. However, their involvement is currently underutilized [42]. Nevertheless, there is a growing recognition that their participation is crucial for the effective implementation of services. For example, targeted education training programs for pharmacist assistants on vaccine screening in addition to the development of standardized screening tools at data entry stations to facilitate identification of vaccination opportunities have proven to be successful in facilitating the provision of immunization services [40,41]. In specific regions of the United States, there are legal provisions allowing pharmacy technicians to administer immunizations [42,43]. In Canada, the province of Ontario has been at the forefront of expanding the responsibilities of pharmacy technicians to encompass the administration of COVID-19 vaccines [44]. Following this trend, other Canadian provinces have also embraced the expansion of the pharmacist’s role, involving pharmacy technicians in administering immunizations. This approach has the potential to enhance workflow efficiency, enabling pharmacists to allocate more time to deliver additional clinical services and engage in discussions related to vaccination status and needs [40,41].

The third competency gap identified in our study pertains to the optimization of workflow and scheduling practices, which is crucial for enhancing the overall efficiency of vaccination services. As previously mentioned, in Canada, pharmacy associations offer training for vaccine administration. However, they do not offer support or guidance on the practical aspects of daily implementation, vaccine promotion, and, most notably, best practices for integrating vaccination services into pharmacy practice and for optimizing staff scheduling. Participants noted that improving workflow and scheduling practices can greatly contribute to reducing wait times and enhancing the overall patient experience, making vaccination services more accessible and appealing to a broader population. During our workshops, pharmacists actively participated in peer-to-peer exchanges, collaborating to develop their own POA, which included optimizing pharmacy scheduling. They considered factors such as overall prescription volume, patient load, staffing requirements, patient profiles, and other unique characteristics of their pharmacy based on its type and location. Of interest, all participants also felt that they required continued assistance for integration of vaccinations into their workflow. Commonly perceived barriers included limited system factors such as inadequate time and insufficient staffing, and identification of vaccine-eligible patients.

The developed framework of best practices encompassed the following key elements: (1) clarifying roles and responsibilities, (2) emphasizing the significance of team meetings to establish shared objectives, (3) implementing reminder strategies such as pharmacy posters, age-based reminder cards included in patients’ prescription bags, or readily available documentation enabling pharmacy staff to identify vaccination opportunities during patient counseling or prescription drop-off, and (4) enhancing staffing and scheduling arrangements on clinic days to effectively meet the heightened demand.

Following the workshop, a total of 66% participants completed the post-workshop evaluation survey (n = 33). Of these, ~90% of the participants (i.e., 29/33) acknowledged that the workshop had a direct influence on their practice through the development of actionable items for them to implement beyond the workshop. Similarly, ~90% of the participants (i.e., 29/33) also felt that the workshops achieved the main goal of developing personalized POAs to optimize vaccination services at their respective pharmacies. When asked to identify the strengths of the activity, a majority of participants expressed their enjoyment of the small group discussions. They found it particularly beneficial as it fostered active participation from all individuals involved. Common themes included appreciating the interactivity and organization of the workshop, in addition to being able to exchange information amongst likewise professionals. This outcome is not surprising, given the extensive evidence from numerous studies showcasing the advantages of small group, interactive, case-based discussions. These discussions have consistently been shown to improve patient outcomes in the pharmacy setting [45,46,47]. Additionally, the chance to participate in discussion-based workshops focused on clinically relevant cases, discussions, and reflections, like the one we conducted, provides a platform for building connections among individuals who share similar goals.

One limitation of our current workshops was the lack of evaluation or incorporation of perspectives from pharmacy assistants and other support staff involved in community pharmacy vaccination services. As a result, we are currently in the process of creating additional workshops specifically tailored to empower pharmacy technicians, pharmacy assistants, and other supportive personnel. The primary objective of these upcoming workshops is to solicit staff feedback and hopefully enhance their proficiency and expertise in supporting immunization services. By doing so, we strive to strengthen the overall capacity of the pharmacy workforce and enhance the accessibility and quality of immunization services provided. Due to the overall success of these workshops, our future objectives also involve collaborating with pharmacists in Canada and elsewhere to explore other emerging topics beyond vaccinations. These include enhancing community pharmacist-led interventions for patients with diabetes [48], optimizing patients’ prescription programs through deprescribing [49], and pharmacist involvement in the screening and management of asthma care and chronic respiratory diseases [50,51]. In conclusion, as the roles of pharmacists and community pharmacies continue to evolve in Canada and across the globe, ensuring successful patient-centered care necessitates enhancing pharmacists’ knowledge, skills, and performance. For the successful adoption of new clinical initiatives, educational programs play a crucial role in updating practitioner knowledge and addressing perceived barriers through strategic solutions.

## Data Availability

The data that support the findings of this study are available on request from the corresponding author. We encourage interested readers to directly contact the corresponding author to obtain workshop materials or to explore opportunities for collaboration in adapting these workshops for different regions. The data is not publicly available due to privacy and copyright restrictions.

## Supplementary Materials

The following supporting information can be downloaded at: https://www.mdpi.com/article/10.3390/pharmacy11040129/s1, Supplementary File S1: Workshop Evaluation Questionnaire; Supplementary File S2: Results from Workshop Evaluation Questionnaire.
