# Supplementary material for: Development and Implementation of Workshops to Optimize the Delivery of Vaccination Services in Community Pharmacies: Thinking beyond COVID-19
Source: Pharmacy (Basel). 2023 Aug 13;11(4):129. doi: 10.3390/pharmacy11040129 (PMC10458354; doi:10.3390/pharmacy11040129)
Supplement: Supplementary file 1 [file pharmacy-11-00129-s001.zip › pharmacy-2484066-supplementary.pdf]

Supplementary Files To:

Development and implementation of workshops to optimize the delivery of vaccination services in community pharmacies: Thinking beyond COVID-19

Corresponding Author:

Arnaud Lavenue, PharmD, MSc

Toc Toc Communications, 104-7030 Rue Marconi, Montréal, Québec, Canada H2S 3K1

Email: alavenue@toctoccommunications.com

Tel: +1 (514) 813-0769

Fax: +1 (514) 868-0815

August 13, 2023

Supplementary File 1:
Workshop Evaluation Questionnaire

1- Very low (VL) **2-** Low (L) **3-** Medium (M) **4-** High (H) **5-** Very high (VH)

**Questions**	**1 VL**	**2 L**	**3 M**	**4 H**	**5 VH**	**N/A**
1. This activity will have a direct impact on my practice.						
2. The workshop allowed me to reach my main goal of developing a personalized action plan to optimize my vaccination service.						
3. will be recommending this activity to other pharmacist-owners.						
4. The content was organized in a coherent and consistent way.						
5. The workshop facilitator interacted with participants.						
6. The discussions I had with colleagues were relevant.						

Supplementary File 2:
Results from Workshop Evaluation Questionnaire

	Responses					
**Question**	**Very Low**	**Low**	**Medium**	**High**	**Very High**	**Total**
Q1	0	1	3	13	16	33
Q2	0	0	4	13	16	33
Q3	0	2	1	13	17	33
Q4	0	1	1	20	11	33
Q5	0	0	0	6	27	33
Q6	0	0	2	12	19	33
